# Supplementary material for: New insight on some selected nanoparticles as an effective adsorbent toward diminishing the health risk of deltamethrin contaminated water
Source: PLoS One. 2021 Nov 4;16(11):e0258749. doi: 10.1371/journal.pone.0258749 (PMC8568195; doi:10.1371/journal.pone.0258749)
Supplement: Attachment — Submitted filename: Response to Reviewers.docx [file pone.0258749.s001.docx]

**Response to Reviewers**

Dear academic editors and reviewers, Thank you very much for your valuable comments that improve the manuscript.

Reviewer #1: The manuscript deals with the "removal of insecticide with adsorption methods (nanoparticles adsorbents).

The movement of pesticides into water bodies can occur through sub-surface drainage, leaching, run-off and spray drift. Hence, the elimination of pesticide residues from the environment has gained widespread attention. Insecticides are broadly applied to control insect pests, but their environmental safety has been a serious concern. The global insecticide market accounted for $14.51 billion in 2016, and it is forecasted to reach $19.27 billion by 2022.

**1.Page 2**; "several kinetic models at pH 7, 40 ppm and 25 °C." It should be corrected as "several kinetic models at pH 7, initial concentration of deltamethrin 40 ppm and temperature 25 °C.

**Author response:** Thank you very much, the required correction was done at page 2

**2. Page 2,** "with removal of 27.56 mg/g, 17.60 mg/g, and 3.06 mg/g of Zn-Al LDH/GA, Zn-Fe LDH, and Fe Oxide, respectively."?! What's the meaning here? It means:
deltamethrin removal of 27.56 mg/L, 17.60 mg/L, and 3.06 mg/L with the Zn-Al LDH/GA, Zn-Fe LDH, and Fe Oxide, respectively.
OR
in deltamethrin removal, the maximum adsorption capacity was 27.56 mg/L, 17.60 mg/L, and 3.06 mg/L with the Zn-Al LDH/GA, Zn-Fe LDH, and Fe Oxide, respectively.

**Author response:** (in deltamethrin removal, the maximum adsorption capacity was 27.56 mg/L, 17.60 mg/L, and 3.06 mg/L with the Zn-Al LDH/GA, Zn-Fe LDH, and Fe Oxide, respectively) is the correct one.

**3.** Effects of pH on adsorption of insecticide should be testes, as an important factor in removal of organic pollutants. I suggest to use a wide range of pH (i.e. 3 to 9).

**Author response:** the effect of pH on adsorption process was described at page 15 & 21 and figure 4.

**4.** Testing adsorption isotherms, Langmuir and Freundlich, are recommended.

**Author response:** Adsorption isotherm models were explained through Langmuir and Freundlich models at page 15 &21, Table 2, figure 5

**5.** The main removal mechanism? electrostatic interactions? van der Waals?

**Author response:** The mechanism were investigated through the kinetic study, FTIR and XRD technique where the adsorption explained by electrostatic attraction and the interaction between the metal ions present in nanomaterials and deltamethrin at page 13, 14 & 22
